# Supplementary material for: Comparative Study of Crystallization, Mechanical Properties, and In Vitro Cytotoxicity of Nanocomposites at Low Filler Loadings of Hydroxyapatite for Bone-Tissue Engineering Based on Poly(l-lactic acid)/Cyclo Olefin Copolymer
Source: Polymers (Basel). 2021 Nov 9;13(22):3865. doi: 10.3390/polym13223865 (PMC8619963; doi:10.3390/polym13223865)
Supplement: Supplementary file 1 [file polymers-13-03865-s001.zip › polymers-1385106-supplementary.pdf]

Article

Comparative Study of Crystallization, Mechanical Properties, and In Vitro Cytotoxicity of Nanocomposites at Low Filler Loadings of Hydroxyapatite for Bone-Tissue Engineering Based on Poly(L-lactic Acid)/Cyclo Olefin Copolymer

Farzana Nazir [1] and Mudassir Iqbal [1,*]

Department of Chemistry, School of Natural Sciences, National University of Science and Technology (NUST), Islamabad 44000, Pakistan; farzana.nazir@sns.nust.edu.pk
* Correspondence: mudassir.iqbal@sns.nust.edu.pk; Tel.: +92-51-9085-5575

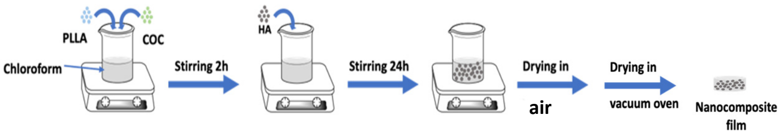

Figure S1. Preparation of PLLA/COC-nHA by physical blending method.

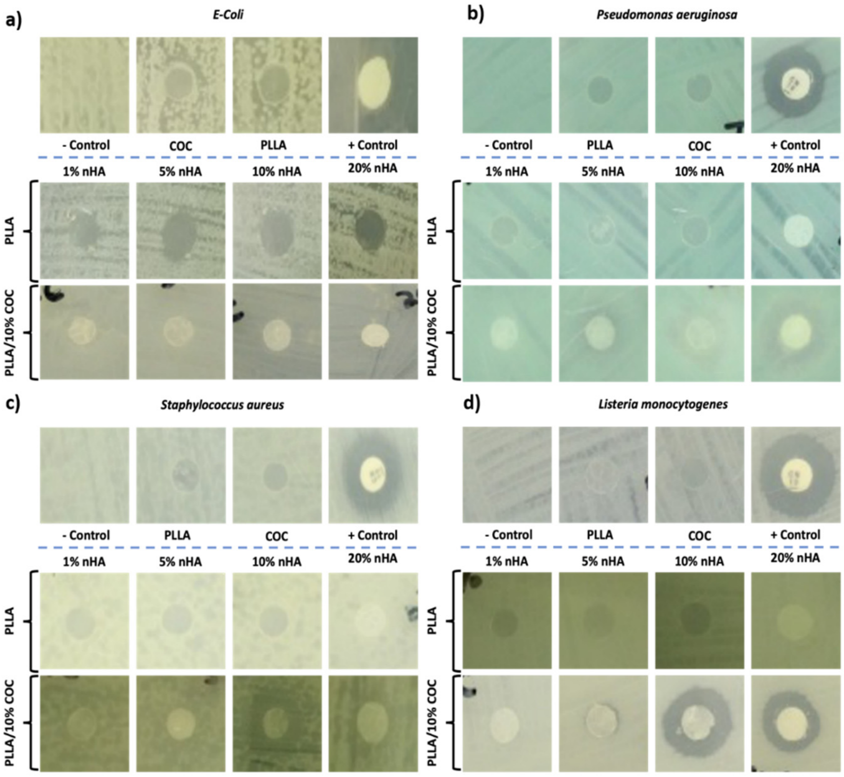

Figure S2. Antimicrobial activity of PLLA, COC, PLLA/nHA, PLLA/10% COC/nHA with (**a**) *E. Coli*, (**b**) *Pseudomonas aeruginosa*, (**c**) *Staphylococcus aureus* and (**d**) *Listeria monocytogenes.*

Table S1. PLLA/COC-nHA and PLLA-nHA nanocomposites composition prepared in 40 mL of Chloroform.

Polymer System	Sample Code	Weight of PLLA (g)	Weight of COC (g)	Weight of HA (g)
PLLA/COC-10	PC10	0.90	0.10	-
PLLA/COC-10-1wt%nHA	PC10-HA1	0.90	0.10	0.01
PLLA/COC-10-5wt%nHA	PC10-HA5	0.90	0.10	0.05
PLLA/COC-10-10wt%nHA	PC10-HA10	0.90	0.10	0.10
PLLA/COC-10-20wt%nHA	PC10-HA20	0.90	0.10	0.20
PLLA	PLLA	1.00	-	-
PLLA-1wt%nHA	P-HA1	0.99	-	0.01
PLLA-5wt%nHA	P-HA5	0.95	-	0.05
PLLA-10wt%nHA	P-HA10	0.90	-	0.10
PLLA-20wt%nHA	P-HA20	0.80	-	0.20
